# Selenium biofortification in conditions of soil salinity improve some nutritional value parameters of spinach for the fresh market

**DOI:** 10.1038/s41598-026-55268-4

**Published:** 2026-06-12

**Authors:** Ewelina Grochowska, Marcin Niemiec, Monika Komorowska, Monika Kula-Maximenko, Tan Suat Hian, Abzal Abdramanov, Anna Gorczyca

**Affiliations:** 1 Department of Agricultural and Environmental Chemistry, Faculty of Agriculture and Economics, University of Agriculture in Krakow, Mickiewicza 21, Krakow, 31-120 Poland; 2 Franciszek Górski Institute of Plant Physiology Polish Academy of Sciences, Niezapominajek 21, Krakow, 30-239 Poland; 3 Faculty of Industrial Sciences and Technology, Universiti Malaysia Pahang Al-Sultan Abdullah, Lebuh Persiaran Tun Khalil Yaakob, Kuantan Gambang, Pahang, 26300 Malaysia; 4 Kazakh National Agrarian Research University, 8, Abay av, Almaty, 050010 Kazakhstan; 5 Department of Microbiology and Biomonitoring, Faculty of Agriculture and Economics, University of Agriculture in Krakow, Mickiewicza 21, Krakow, 31-120 Poland

**Keywords:** Soil, Salinity, Spinach, Selenium, Biofortification, Yield, Biochemistry, Environmental sciences, Plant sciences

## Abstract

This study investigated the interactive effects of soil salinity (2.88 and 3.79 dS⋅m^− 1^) and foliar sodium selenate application (0.80 and 0.15 kg Se⋅ha⁻^1^) on the yield parameters and phytochemical composition of spinach (*Spinacia oleracea* L. cv. Matador) under the conditions of semi-controlled pot trial under field conditions. The experiment confirmed that selenium biofortification is effective, increasing its levels in the plant ten-fold. Selenium has a positive effect, mitigating salt stress, and its uptake is closely linked to sulfur metabolism. Foliar application of selenium to spinach under salt stress conditions (up to 3.79 dS⋅m^− 1^) does not negatively impact yield, effectively increasing the content of bioactive components such as phenols and antioxidants. Studies confirm the synergistic effects of selenium and salinity, improving stress tolerance, mitigating sodium toxicity, and stimulating dry matter growth and S/Zn/Mn content. These findings demonstrate the viability of selenium foliar application for producing nutritionally enhanced spinach on salt-affected agricultural soils.

**Supplementary Information:**

The online version contains supplementary material available at 10.1038/s41598-026-55268-4.

## Introduction

Contemporary agricultural research increasingly focuses on soil degradation as the primary constraint limiting sustainable crop production systems^1^. Mitigation of technology-induced soil deterioration is a critical prerequisite for maintaining long-term agricultural productivity while preserving ecosystem integrity^2^. Secondary salinization, resulting from intensive agricultural practices, industrial activities, and mining operations, has emerged as a widespread threat to agroecosystem functionality, necessitating adaptive cultivation strategies for salt-affected soils.

Soil salinization affects approximately 424 million hectares of topsoil (0–30 cm in depth) and 833 million hectares of subsoil (30–100 cm in depth) globally, placing a critical constraint on agricultural productivity^3^. Current estimates indicate that salinization impacts over 1 billion hectares of agricultural land, accounting for more than 20% of irrigated cropland worldwide^4^. Climate change-induced phenomena, including sea-level rise, altered precipitation patterns, and increased frequency of extreme weather events, exacerbate salinization processes in coastal regions and previously unaffected inland areas^5,6^.

Soil salinity occurs when concentrations of soluble salts (primarily NaCl, Na_2_SO_4_, and NaHCO_3_) exceed plant tolerance thresholds, typically defined as electrical conductivity > 2 dS⋅m⁻^1^. The primary physiological consequence involves osmotic stress, which reduces water potential gradients and impairs cellular water uptake, creating conditions analogous to drought stress^7^. Secondary effects include ion-specific toxicity, particularly sodium-induced displacement of essential cations (K⁺, Ca²⁺, Mg²⁺), chloride-mediated inhibition of photosynthetic processes, and oxidative stress resulting from enhanced reactive oxygen species production^8,9^.

Global food security paradigms have evolved beyond caloric adequacy to encompass micronutrient density and bioavailability in staple food crops. Micronutrient malnutrition, termed “hidden hunger,” affects an estimated 2 billion individuals worldwide, with deficiencies in zinc, iron, selenium, iodine, and essential vitamins being the most prevalent nutritional inadequacies^10^. Plant-based diets, while environmentally sustainable, often exhibit suboptimal micronutrient profiles, particularly for selenium, zinc, and vitamin B_12_, necessitating targeted biofortification strategies^11,12^.

Green leafy vegetables are growing in popularity, largely because they are considered an important part of a healthy diet and a significant component of the widely practiced vegetarian and vegan diets. Numerous studies indicate that increased consumption of green leafy vegetables can provide the nutritional requirements necessary for proper growth as well as adequate protection against diseases caused by malnutrition^13^. Leafy vegetables can be the cheapest and most readily available source of essential nutrients, including vitamins, minerals, amino acids, and other compounds that help maintain health, thus counteracting hidden hunger^14^.

From the point of view of growing crops for direct consumption, especially green leafy vegetables, hydration is an important parameter. Saline substrate interferes with water uptake by plants and decreases tissue hydration^15^. An important effect of substrate salinity is also reduced health values of leafy vegetables^16^.

*Spinacia oleracea* L. is a nutrient-dense leafy vegetable characterized by high concentrations of folate, β-carotene, lutein, nitrates, and phenolic compounds, conferring significant antioxidant, anti-inflammatory, and cardioprotective properties^17^. The species exhibits rapid growth kinetics (harvest maturity: 35–45 days), broad thermal tolerance (optimal: 15–18 °C), and moderate soil pH requirements (6.0–7.5), making it suitable for intensive production systems^18^. However, spinach demonstrates limited salt tolerance (threshold: ~2–3 dS⋅m⁻¹), with salinity stress significantly reducing leaf quality parameters essential for fresh market applications.

One method of increasing plant resistance to increased soil salinity is to biofortify plants with selenium or zinc, for example, something which has already been tried in the scientific research^19–21^. However, there is a lack of comprehensive studies on the effects of foliar-applied selenium not only on plant yield but also on the chemical composition of spinach plants that would take into account not only content in mineral nutrients, phenolic compounds and vitamins that determine the nutritional quality of spinach under conditions of increased soil salinity.

This investigation aimed to quantify the individual and interactive effects of soil salinity stress (3 and 6 mmol NaCl⋅dm⁻^3^) and foliar selenium biofortification (0.80 and 0.15 kg Se⋅ha⁻^1^ as Na_2_SeO_4_) on morpho-physiological parameters, mineral composition, and bioactive compound profiles in *S. oleracea* cv. Matador. Specific objectives included evaluation of the following: (1) leaf yield and dry matter content; (2) content in photosynthetic pigments and L-ascorbic acid, as well as parameters of the antioxidant systems; (3) selenium accumulation; (4) the leaf elemental composition after the applied treatments.

## Materials and methods

### Experimental design

A two-factor vegetation experiment was conducted in a commercial company that engages in large-scale spinach production for the European market. The experimental plant was spinach cv. Matador. The soil used in the experiment had a granulometric composition and the parameters shown in Table 1.

**Table 1:** Evaluated properties of the soil used in the pot experiment.

Parameter		Unit	Value
Granulometric composition	sand	%	44
	coarse dust		21
	fine dust		11
	coarse clay		18
	colloidal clay		6
pH KCl			5.72
pH H_2_O			6.38
Chemical composition	total N	%	0.904
	organic C		0.970
	mineral N	g⋅kg^− 1^	0.0492
	P		0.1264
	K		0.2315
	Mg		0.1894
	Ca		0.4763
	Cl		0.0283
	Na		0.0235
	S-SO_4_		0.0136

Granulometric composition was determined by sedimentation, using the Bouyoucos-Casagrande areometric method modified by Proszyński^22^. Electrical conductivity (EC) was determined conductometrically. Reactivity was determined by the potentiometric method. Organic carbon and total nitrogen content were determined by elemental analysis using an Elementar Variomax Cube apparatus. Mineral nitrogen content was determined by the distillation method after previous extraction with potassium sulfate solution of 1 mol⋅dm^− 3^^23^. The content of bioavailable forms of elements (P, K, Mg, Ca, Cl, Na) was determined by inductively coupled plasma optical emission spectrometry (ICP-OES) using an Optima 7600 spectrometer (PerkinElmer, USA) after extraction with Mehlich 3 solution^24^. S-SO_4_ content was determined by ICP-OES (as above) after extraction with acetic acid^25^.

Meteorological conditions such as average daily air and soil temperature; dew point and DeltaT (wet and dry bulb temperature difference); relative humidity and daily precipitation; wind speed, solar radiation and ET0 (sum of water evaporation from the soil and water transpiration through plants) during the spinach growing season are presented in the supplementary information file. During the spinach growing season, conditions were moderately favorable. Air and soil temperatures were within the range favorable to growth, although in the latter part of the growing season they approached the upper limit of the optimum range, which could have accelerated plant senescence. The main deviations concerned water conditions – rainfall was very low, and evapotranspiration (ET0) and water vapor deficit (VPD) values were often high. Low relative humidity meant a risk of drought stress, so maintaining optimal conditions required regular irrigation. The initial total water capacity used in the experiment was 60%. Constant humidity was maintained by weighing the pots daily and topping them up with distilled water. Overflowing was avoided to prevent electrolyte loss.

The vegetation experiment was conducted in a randomized complete block design (RCBD) with four replications (blocks). The blocking was used to control environmental variability. The experimental unit was a 300 mm diameter pot containing 10 kg of soil. Twelve spinach seeds were sown in each pot. After two weeks of growth, seven uniform, healthy plants were left per pot. All measurements were performed for each pot separately: yield was determined as plant biomass from the entire pot (converted to t⋅ha^− 1^ using the density calculation in the cultivation of Matador spinach i.e. 1 mln plants per hectare), while biochemical parameters were determined from leaf samples taken from all plants in the pot and then averaged. Therefore, each pot constituted an independent experimental unit. The total number of pots was 36 (9 treatment combinations × 4 replicates). The experimental factors were: salt stress (NaCl) - applied by fertigation at three levels: (control 0 mmol NaCl·dm⁻³, low stress 3 mmol NaCl·dm⁻³, high stress 6 mmol NaCl·dm⁻³). Salinity levels were selected based on literature on the sensitivity of *S. oleracea* to salt stress^26^. The moderate tolerance of spinach suggests that a concentration of 3 mmol NaCl·dm⁻³ represents mild stress conditions, while a concentration of 6 mmol NaCl·dm⁻³ induces physiological changes related to excess Na and Cl ions, allowing the plant’s defense response to be assessed under controlled fertigation conditions. The NaCl concentration in the working solution was 35 mmol NaCl·dm⁻^3^, and the amount of salt used was controlled by the application rate. The NaCl dose was divided to avoid osmotic shock. The soil in the objects without salinity was characterized by an EC value of 0.23 dS⋅m^− 1^, and the salinity of the soil solution under actual conditions, while maintaining the moisture content at 60% of the total water capacity of the soil, was 2.88 dS⋅m^− 1^ at the lower salinity level and 3.79 dS⋅m^− 1^ at the higher salinity level.

Selenium fertilization - applied foliarly in the form of sodium selenate (Na₂SeO₄) at three levels: (control 0 kg Se·ha⁻¹, low dose 0.08 kg Se·ha⁻¹, high dose 0.15 kg Se·ha⁻¹). The working solution for foliar application was acidified with citric acid to pH 5.5.

Selenium fertilization and salt stress were applied twice, 7 days apart. The experimental design was a full factorial 3 × 3 (NaCl levels × Se levels) setup, resulting in nine treatment combinations: control (0/0), low salinity (3/0), high salinity (6/0), low selenium (0/0.08), high selenium (0/0.15), low salinity + low selenium (3/0.08), low salinity + high selenium (3/0.150), high salinity + low selenium (6/0.08), and high salinity + high selenium (6/0.15). The scheme of the treatments carried out and the determinations of the treatment data are shown in Table 2. Plant growth lasted for 5 weeks after the second fertigation treatment, after which the evaluation began.

**Table 2:** Experimental scheme.

Object designation	Abbreviation	NaCl dose [mmol⋅dm^− 3^] of soil	electrical conductivity (EC) [dS⋅m^− 1^] of soil	Se dose [kg⋅ha^− 1^] of crop
Control	control	0	0.23	0
Low salt stress	SL	3	2.88	0
High salt stress	SH	6	3.79	0
Selenium low dose	SeL	0	0.23	0.08
Selenium high dose	SeH	0	0.23	0.15
Low salt stress plus selenium low dose	SLSeL	3	2.88	0.08
Low salt stress plus selenium high dose	SLSeH	3	2.88	0.15
High salt stress plus selenium low dose	SHSeL	6	3.79	0.08
High salt stress plus selenium high dose	SHSeH	6	3.79	0.15

The fresh weight yield of spinach plants per hectare of cultivation was evaluated first. Dry matter content was determined by drying to constant weight at 65 °C. Ascorbic acid content was determined by titration^27^. Spinach leaves were homogenized with 1% oxalic acid. The determination was based on the oxidation reaction in acid medium of L-ascorbic acid to dehydroxyascorbic acid using 2,6-dichlorophenolindophenol. Excess pigment in the acidic medium gives a pink color and marks the end point of the titration. The content of ascorbic acid in the sample was calculated from the known amount of modified 2,6-dichlorophenolindophenol solution used for titration. The content of photosynthetic pigments (chlorophyll *a*, chlorophyll *b*, carotenoids) was determined by spectrophotometric method in a Beckman DU 640 UV-VIS spectrometer (Beckman Coulter, USA). The content of pigments was calculated from absorbance readings at wavelengths of λ = 663, λ = 646, λ = 470, respectively, using molar absorbance ratios. Antioxidant activity was determined by the method of Brand-Williams et al.^28^ using the DPPH (2,2-diphenyl-1-picrylhydrazyl) radical. The method is based on the reduction of the DPPH radical by antioxidants in the plant material. The solution of the radical changes color from violet to yellow, and the decrease in absorbance over time is measured in a UV-VIS spectrophotometer, at a wavelength of λ = 517 nm, against a control solution. Antioxidant activity was expressed as the % of DPPH reduced by the antioxidants present in the plant material. Total phenolic content was determined by the Folin-Ciocalteu method using gallic acid as standard. Phenolic compounds were extracted from plant tissues with 80% methanol solution. The determination is based on the reversible reduction reaction of phenols in an alkaline medium from molybdenum(VI) to molybdenum(V) contained in the Folin-Ciocalteu (F-C) reagent. The reaction produces a blue compound with an absorption maximum at λ = 745–750 nm. The intensity of absorption at this wavelength is proportional to the concentration of phenols in the sample. The macro- and microelement content of the plant material was determined by the ICP-OES method (as above) after prior wet mineralization in a closed system. An Anton Paar Multiwave 5000 microwave mineralizer was used for mineralization.

All chemical analyses were performed in three technical replicates, and the mean of these replicates was used for further statistical analysis.

### Statistical calculation and data analysis

Data was analyzed using two-way ANOVA with soil salinity and selenium biofortification as fixed factors. Prior to analysis, assumptions of normality and homogeneity of variances were checked. Normality of residuals was assessed using the Shapiro–Wilk test and Q–Q plots, while homogeneity of variances was evaluated by Levene’s test. Data analysis, graphing and principal component analysis (PCA) were performed using Origin(Pro), Version 2020 (OriginLab Corporation, Northampton, MA, USA).

## Results

### Effect of selenium biofortification on spinach biomass under salt stress

**Fig. 1:**
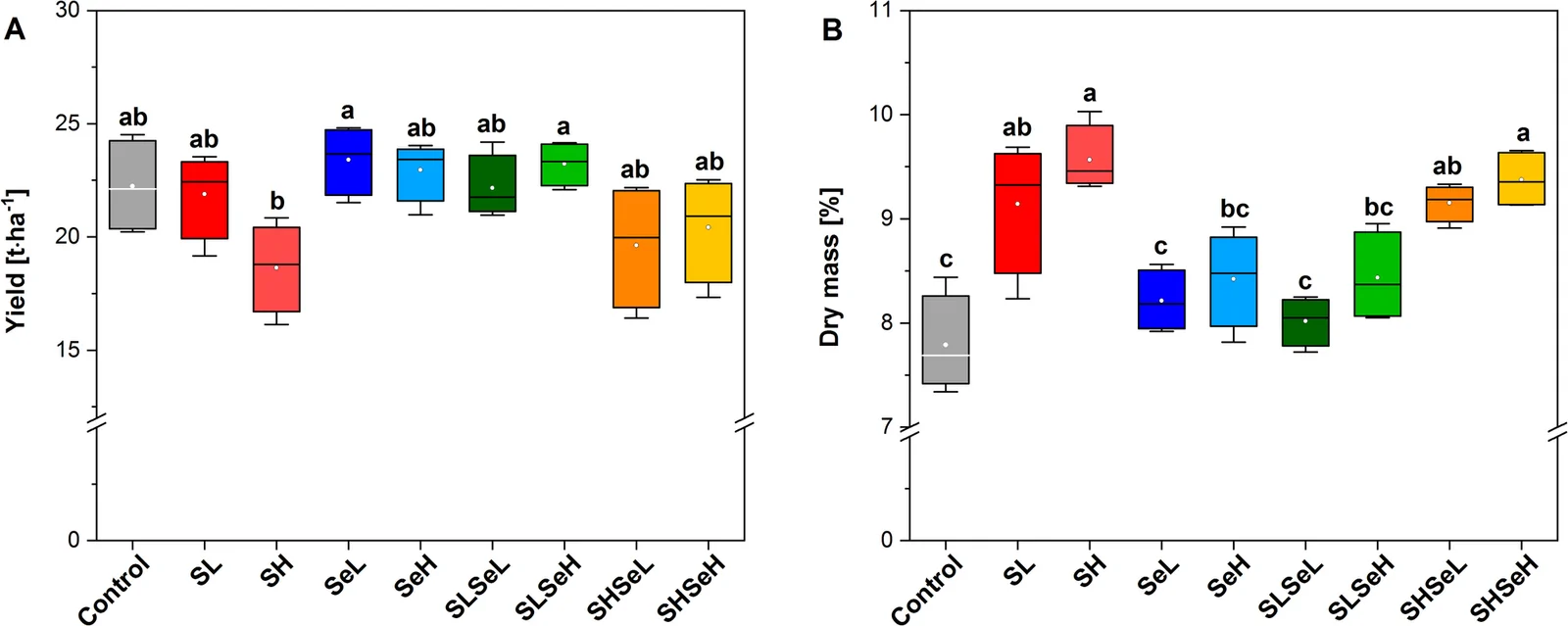
Effect of spinach treatment on fresh leaf biomass yield (**A**) and dry matter bioaccumulation (**B**). Treatment variants are shown on the horizontal axis according to Table 2. Letters refer to all-pairwise comparisons; means not sharing a letter are significantly different (*p* < 0.05).

The post-hoc analysis of detailed comparisons showed that Se biofortification did not significantly affect fresh biomass accumulation across treatments and the control (Fig. 1A), indicating that the applied concentrations remained below phytotoxic thresholds. Mean fresh yields ranged from 18.6 to 23.4 t⋅ha⁻¹, with numerical increases observed under low salinity + high selenium (SLSeH), as well as under low Se dose (SeL) treatment relative to high salinity (SH). Dry matter content exhibited significant salinity effects (Fig. 1B), demonstrating 15–23% higher dry matter accumulation compared to Se biofortification and the control, indicating osmotic adjustment responses typical of glycophytic species under moderate salt stress. A significant interaction of factors was found only for dry matter content (analysis results shown in the supplement). Higher salinity (SH) significantly influenced the higher dry matter accumulation in the yield, which can be explained primarily by the plant response to osmotic stress, the accumulation of compatible osmotic substances, and anatomical adaptation of plants. Se biofortification and higher salinity resulted in higher dry matter accumulation without a significant effect on yield. This confirms that spinach is tolerant to salinity (up to 3.79 dS⋅m^− 1^), allowing spinach to maintain a constant or even slightly higher dry matter yield while adapting to higher salinity.

### Effect of selenium on photosynthetic pigments content in spinach under salt stress

**Fig. 2:**
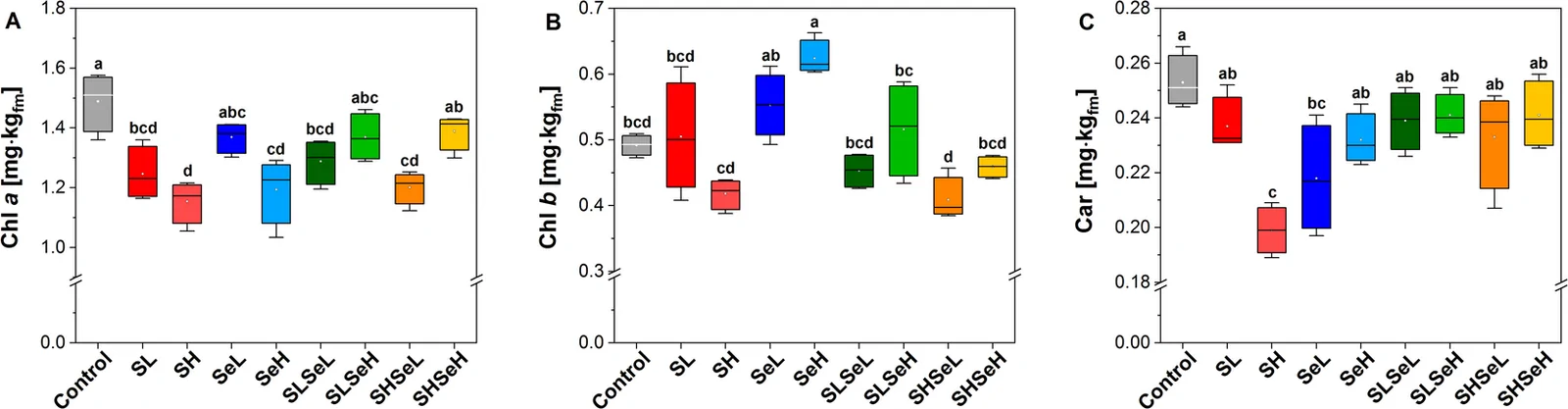
Content of basic photosynthetic pigments in the biomass of spinach crop grown under salt stress and Se biofortification (**A**) Chl *a* = chlorophyll *a*, (**B**) Chl *b* = chlorophyll *b* and (**C**) Car = carotenoids. Treatment variants are shown on the horizontal axis according to Table 2. Letters refer to all-pairwise comparisons; means not sharing a letter are significantly different (*p* < 0.05).

Photosynthetic pigment concentrations showed some significant treatment-dependent variations in the post-hoc analysis of detailed comparisons (Fig. 2). Salinity stress induced dose-dependent reductions in chlorophyll *a* content, with high salinity treatments (SH) exhibiting significant decreases relative to the control. Higher Se biofortification (SeH) improved chlorophyll degradation induced by higher doses of salinity, which was not observed for the lower doses of Se (SeL) used. Carotenoid stability remained largely unchanged except under a combined high salinity + low Se biofortification dose (SHSeL). Results indicating protective interactions in pigments systems between Se biofortification in 0.15 kg⋅ha^− 1^ of crop dose and 3.79 dS⋅m^− 1^ of soil salinity level.

Overall analysis of variance showed a significant decrease in chlorophyll *a* content for the salinity levels used as well as significant interactions - the higher of the factors used are in particular dependent on each other. Chlorophyll *b* accumulation was significantly increased by Se biofortification and reduced by salinity use. The interaction between salinity and Se biofortification was not significant in case of carotenoids. Data is shown in the supplement.

### Effect of selenium on phenolic compounds and ascorbic acid content, and antioxidant activity of spinach under salt stress

**Fig. 3:**
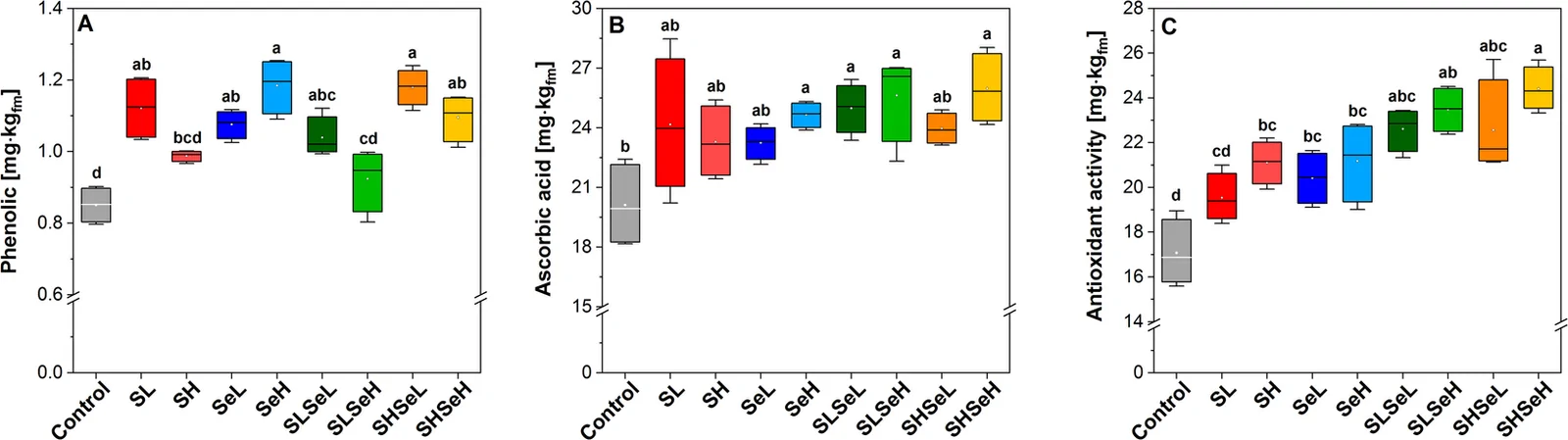
Content of phenols (**A**), L-ascorbic acid (**B**) and antioxidant activity (**C**) of the fresh spinach yield crop grown under salt stress and Se biofortification. Treatment variants are shown on the horizontal axis according to Table 2. Letters refer to all-pairwise comparisons; means not sharing a letter are significantly different (*p* < 0.05).

Bioactive compound post-hoc analysis revealed significant some treatments effects on total phenolic content (Fig. 3A), L-ascorbic acid concentrations (Fig. 3B), and DPPH radical scavenging capacity (Fig. 3C). Selenium biofortification induced increases in phenolic compounds and antioxidant activity, but an increase in L-ascorbic acid content was only seen at the higher Se dose (SeH). Salinity also influenced induction of the plant protective system. Significant increases in L-ascorbic acid levels in saline soil were only seen in the presence of selenium biofortification, potentially reflecting enhanced antioxidant defense mechanisms. Antioxidant capacity, expressed as percent DPPH inhibition, improved significantly in all selenium treatments and treatments with an interaction of factors (35–52% increase), indicating enhanced free radical scavenging potential. Overall analysis of variance showed dependence of factors only for phenols content. Data is shown in the supplement.

### Effect of selenium on spinach elemental composition under salt stress

**Fig. 4:**
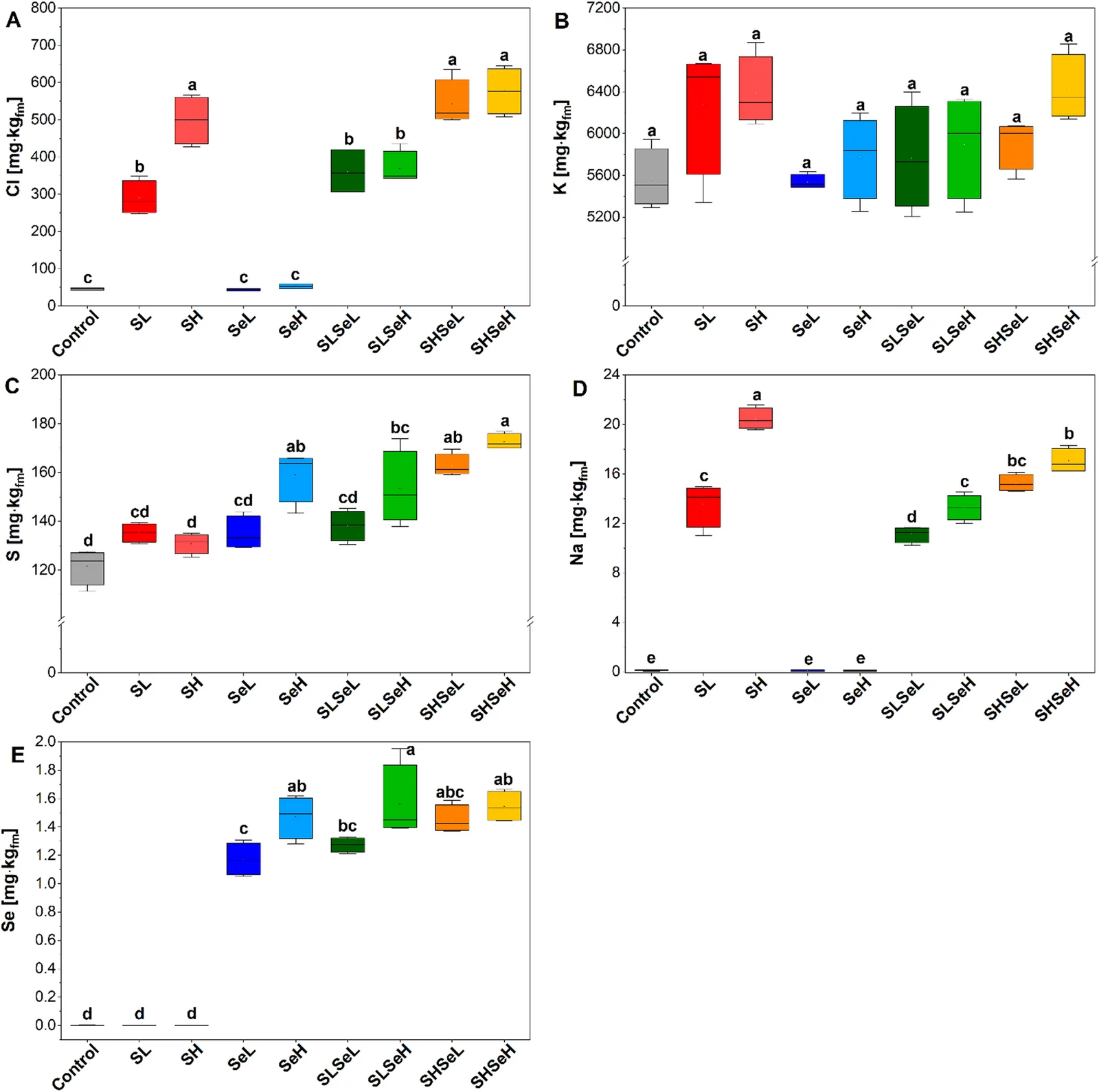
Chemical composition (**A** chlorine, **B** potassium, **C** sulfur, **D** sodium and **E** selenium content) of the fresh spinach yield crop grown under salt stress and Se biofortification. Treatment variants are shown on the horizontal axis according to Table 2. Letters refer to all-pairwise comparisons; means not sharing a letter are significantly different (*p* < 0.05).

ICP-OES analysis confirmed successful selenium biofortification across all treatments, with tissue concentrations ranging from 1.17 to 1.56 mg Se⋅kg⁻^1^ fresh weight, which is a ten-fold increase relative to the controls (0.002 mg Se⋅kg⁻^1^). Selenium recovery efficiency, calculated as absorbed selenium per unit applied, ranged from 21% (SHSeH) to 36% (SHSeL), indicating dose-dependent uptake kinetics (data shown in the supplement). Salinity induced sodium accumulation (3- to 4-times increase) was partially mitigated by selenium biofortification, suggesting competitive uptake mechanisms. Sulfur content increased proportionally with selenium dosage, reflecting shared transport pathways and metabolic integration. Potassium accumulation was not dependent on the treatments used. The overall interaction analysis showed statistical significance only for sulfur content. Figure 4.

Mg, Ca, and Cu contents in the spinach crop at the applied salinity levels and selenium biofortification were not significantly different from the control. Significantly lower Cu concentrations compared to the control were found only in the treatment with a lower dose of selenium biofortification at lower soil salinity (SLSeL). Fe concentrations increased in plants grown under saline soil conditions. Large variations among treatments were observed for Zn and Mn. The increase in the concentration of these micronutrients in treated plants compared to the control was highest for the interaction of higher salinity and Se biofortification levels (SHSeH). Se biofortification did not significantly increase Mn content, but increased Zn content compared to the control. Soil salinity, as well as the interaction of this factor with selenium biofortification, caused an increase in the concentration of both Zn and Mn in the yield biomass compared to the control. The results obtained are shown in Table 3.

**Table 3:** Contents of micro- and macroelements in the fresh mass yield of spinach grown at the applied levels of salinity and selenium biofortification [g⋅kg^− 1^ fresh weight]. Abbreviations of treatment variants as per Table 2.

Treatment	Zn		Mn		Fe		Cu		Mg	Ca
SL	0.00619	abc*	0.00269	cde	0.00199	b	0.00109	ab	0.48	0.84
SH	0.00664	ab	0.00301	cd	0.00238	a	0.00096	ab	0.47	0.83
SeL	0.00593	bc	0.00261	de	0.00143	c	0.00092	ab	0.39	0.76
SeH	0.00602	bc	0.00247	de	0.00155	c	0.00088	ab	0.38	0.75
SLSeL	0.00553	cd	0.00292	cd	0.00164	c	0.00079	b	0.38	0.69
SLSeH	0.00628	abc	0.00322	bc	0.00151	c	0.00091	ab	0.38	0.70
SHSeL	0.00614	bc	0.00364	ab	0.00149	c	0.00102	ab	0.42	0.78
SHSeH	0.00705	a	0.00401	a	0.00149	c	0.00089	ab	0.40	0.78
Control	0.00489	d	0.00220	e	0.00145	c	0.00112	a	0.45	0.77

* Letters refer to all-pairwise comparisons; means in column not sharing a letter are significantly different (*p* < 0.05).

### Principal component analysis of the effect of selenium on spinach composition under salt stress

**Fig. 5:**
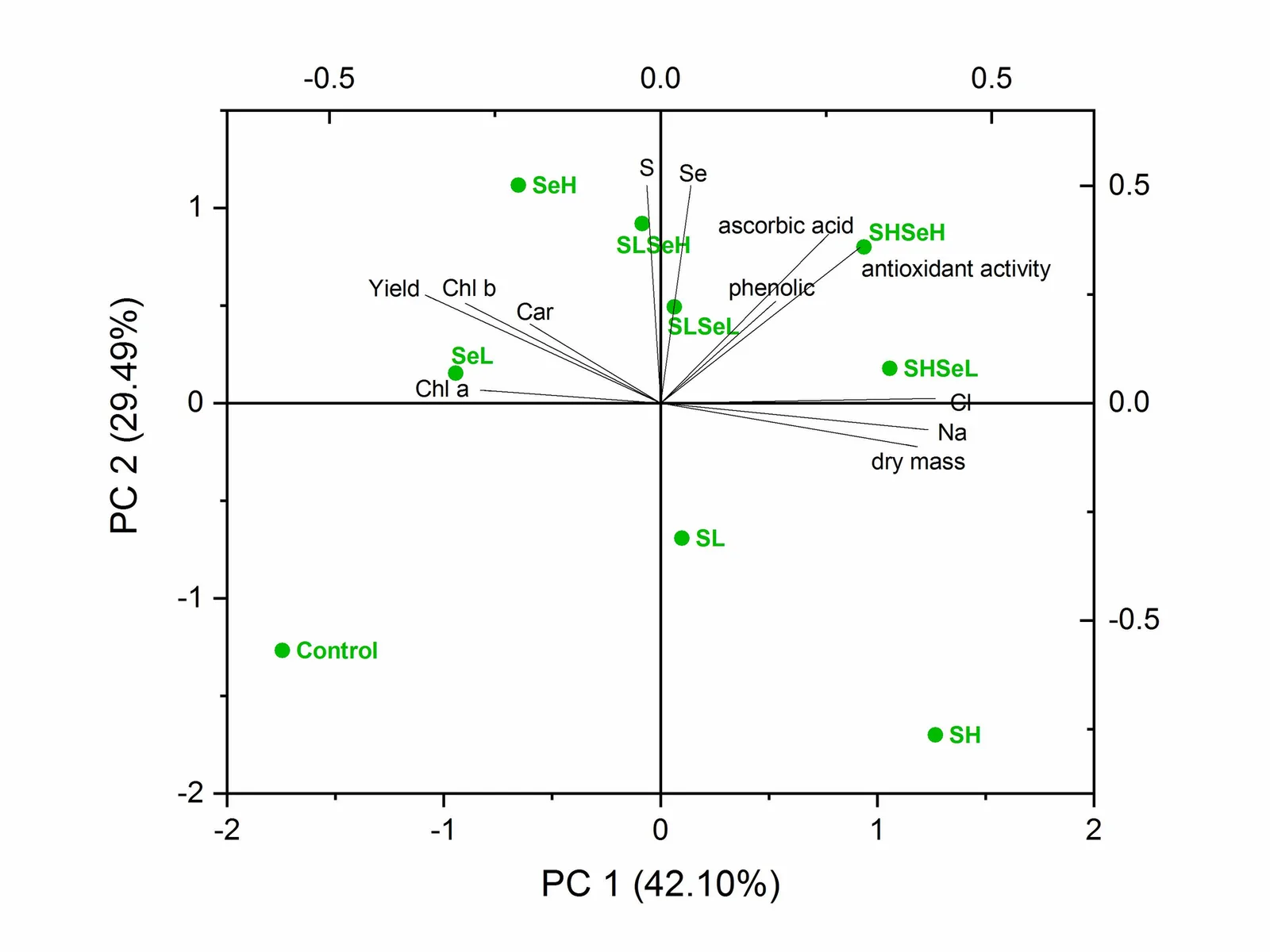
Principal component analysis (PCA) indicates regularities among variables of experimental objects. Treatment variants as per Table 2 shown in green. The abbreviations used for the tested parameters indicate the contents in fresh spinach biomass: Chl a = chlorophyll *a*, Chl b = chlorophyll *b*, Car = carotenoids, S = sulfur, Se = selenium, Cl = chlorine, Na = sodium. Dry mass means percentage of dry matter.

Multivariate analysis via principal component analysis explained 71.59% of total variance across measured parameters (PC1: 42.1%, PC2: 29.49%). Treatment clustering revealed distinct groupings: (1) control samples characterized by baseline nutrient profiles; (2) salinity treatments associated with elevated Na⁺/Cl⁻ content and osmotic adjustment responses; (3) selenium biofortification treatments correlated with enhanced antioxidant compounds and photosynthetic efficiency; (4) interactive treatments (salinity × selenium) exhibiting intermediate characteristics with enhanced stress tolerance markers. Loading analysis identified selenium content, antioxidant capacity, and phenolic compounds as primary discriminating variables. Figure 5.

## Discussion

Plant-based food systems require strategic micronutrient enhancement to address nutritional adequacy, particularly for populations following vegetarian or vegan dietary patterns^12^. Selenium, functioning as a cofactor for glutathione peroxidase, thioredoxin reductase, and selenoprotein P, plays critical roles in antioxidant defense, thyroid hormone metabolism, and immune system regulation^11,29^. In the context of spinach, the primary goal of biofortification is to achieve a selenium level that will meet a significant portion of the recommended daily allowance (RDA) in a standard serving, while not exceeding the toxic threshold. For populations on plant-based diets, the target parameters for spinach include achieving a level of approximately 0.2–0.5 mg Se⋅kg^− 1^ of fresh weight. This allows for nearly 40–90% of the daily allowance (which for adults is approximately 0.55 mg) to be met in a single serving. An additional benefit is conversion into the most bioavailable and safest organic forms for humans, i.e., selenomethionine or methylselenocysteine. Biofortification is an effective method of providing dietary Se, but one should remember about the possible toxicity of excessively high doses. Long-term Se intake exceeding safe level can lead to selenosis, causing hair loss, nail damage, fatigue, and nervous system abnormalities. Intakes of 0.75–0.85 mg daily can lead to functional signs of toxicity^30^.

The Se concentrations in tested spinach tissue observed in our own research were of up to 1.6 mg Se⋅kg^− 1^ of fresh weight even in the case of soil salinity. Biofortification, a process that involves the application of foliar treatments to plants during the growing season with the objective of enhancing the concentration of the applied compound in the crop, has been identified as a potentially efficacious method for leafy vegetables^10,20^. However, the effectiveness of biofortification is influenced by soil pH, redox potential, and the availability of other elements, which complicates the unification of this technology^31^. The conditions used in this study revealed a high Se bioaccumulation potential. This indicates that under these specific conditions, targeted Se biofortification can be effective even at lower spray concentrations.

Biomass analysis revealed a significant increase in Se bioaccumulation with increasing applied dose. A trend toward increased bioaccumulation (slightly higher concentrations) was also observed under saline conditions. The recovery of applied selenium, defined as the ratio of the element absorbed by the crop to the applied dose, ranged from 21% to 36%. It is worth noting that this coefficient was higher at lower selenium doses. This is an important practical guideline. Furthermore, effective and safe Se application rates in the crop may represent a cost-effective biofortification strategy for commercial spinach production, particularly in saline soils where conventional production faces significant limitations. This is important for promoting Se biofortification given the current high cost of Se fertilizers.

Given the Se enrichment of the spinach crop, it is particularly recommended for consumers following a vegan or vegetarian diet, where Se deficiencies, along with those in vitamin B_12_, Zn, and Ca, are identified as significant concerns^12^. In the context of our study, we observed no change in the Ca content of the crop biomass. However, Se biofortification under saline soil conditions led to an increase in Zn and Mn concentrations in the crop. The recommendation for Zn intake is well-established, irrespective of the age of the consumer, for both adults on plant-based diets and omnivorous children^32^. Increasing the Mn content in the tested spinach is also nutritionally beneficial by having an antioxidant effect, regulating sugar metabolism, supporting the metabolism of amino acids, cholesterol and carbohydrates. Mn also supports the skeletal system, as well as thyroid health^33^.

The applied treatments did cause changes in sulfur content. In the context of foliar application, a fundamental concern in soil fertilization with Se – namely, the competition between this element and S due to the similarity in their chemical structure – was not substantiated^34^. Furthermore, soil salinity did not result in S accumulation in yield biomass, nor did a lower Se application rate at lower salinity levels. The elevated S content was predominantly associated with the elevated Se dose utilized for fortification, with soil salinity exhibiting no capacity to restrict this process. The importance of S in enhancing the quality of vegetables and their sensory attributes is well-documented, with studies highlighting its role in increasing soluble sugars and proteins^35^. Furthermore, S, as a biogenic element, contributes to optimal plant growth and maintains homeostasis of essential micronutrients (Mo, Mn, Zn, Fe, Cu), which are crucial for metabolic processes in plants, including protein synthesis, photosynthesis, respiration, DNA synthesis, and the electron transport chain. Based on literature-supported assessments, higher S content in food, as well as Se, can be considered to be particularly beneficial for plant-based diets, where deficiencies of this element are found^36^.

Soil salinity resulted in a substantial augmentation of chlorine content within the crop biomass, with Se biofortification demonstrating no significant impact in this instance. The potential impact of Se on Cl bioaccumulation is corroborated by Gui et al.^37^. In addition, bioaccumulation of sodium in the crop was found to be contingent on the level of salinity, with growing crops on saline substrates generally resulting in increased Na content in plant biomass^38^. Our study demonstrated that Se biofortification significantly reduced Na content.

The Se biofortification led to enhanced phenolic content and antioxidant activity, which remained consistent in the presence of salinity. Spinach is a vegetable with a particularly high content of phenolic compounds and high antioxidant activity^39^. Numerous scientific papers have confirmed the significant health benefits associated with the consumption of foods rich in phenols and with high antioxidant activity. These benefits include a reduction in the incidence of metabolic diseases, including diabetes, hypertension, neurodegenerative diseases, cardiovascular diseases, and obesity^40–42^.

Ascorbic acid, a compound that is found in all plant species and tissues, plays a pivotal role in various physiological processes, including energy metabolism, growth, development, and response to biotic and abiotic stresses. It is also a nutritionally beneficial compound. Siener et al.^43^ found that, when consumed, spinach rich in ascorbic acid can counteract the negative effects of oxalic acid, a nutritionally unfavorable compound. However, the underlying mechanisms by which ascorbic acid and oxalic acid are metabolized in edible vegetables remain to be fully elucidated^44^.

It was found that the applied levels of soil salinity and Se biofortification, both separately and in combination, did not exert a significant effect on the amount of fresh spinach biomass yield. Consistent with these findings, Ors and Suarez^45^ and Ferreira et al.^46^ reported similar outcomes for the salinity factor, indicating that irrigating spinach with water containing a salinity of 9–10 dS⋅m^− 1^ did not have a negative impact on yield. A comprehensive review by Moulick et al.^47^ underscores the significance of Se fertilization in enhancing dry matter yield under abiotic stress conditions, including soil salinity. Zhang et al.^48^ found that Se, by regulating the opening and closing of stomata, can contribute to reducing evapotranspiration, which is beneficial, especially in cases of limited water supply and increased soil salinity. In contrast, our study demonstrated that dry matter accumulation was significantly higher under conditions of salinity, a finding that was not observed in the context of Se biofortification. However, it is crucial to emphasize that, for leafy vegetables intended for the fresh market, leaf water content is a pivotal quality parameter, a distinction from typical agricultural crops. The salinity levels used in this study exerted an adverse effect on the water content of the crop. However, Se biofortification at lower salinity levels negated this effect, a finding that was not observed at higher levels.

The study confirms the findings that Se foliar application is more efficacious, offering a straightforward method to enhance biofortification, thereby increasing Se concentrations without compromising yields, in contrast to soil application, where effectiveness is contingent on soil chemistry, microbial activity, and oxygenation levels^49^. However, it is imperative to acknowledge the narrow boundary between biofortification and the toxic effects of Se, which is contingent on the element’s concentration, its chemical form, the method of application, and the plant genotype^50,51^. Furthermore, environmental factors, including soil pH, the sulfur/selenium ratio, rainfall intensity, and temperature, can influence Se uptake, accumulation, and its subsequent biological activity in plants. This complexity renders the establishment of universal guidelines for phytofortification impractical.

## Conclusions

This study demonstrates the technical feasibility and agronomically viable of Se biofortification in *S. oleracea* cv. Matador under moderate salinity stress. Key findings include: (1) successful biofortification with selenium (1.17–1.56 mg Se⋅kg^⁻1^) with recovery rates ranging from 21% to 36%; (2) improved antioxidant profiles and phenolic compound concentrations due to selenium-salinity interaction; (3) improved nutritional value, particularly of sulfur, zinc and magnesium, in the crop biomass; and (4) synergistic effects of selenium and salinity, boost stress tolerance and mitigating sodium toxicity. These results provide a basis for implementing Se biofortification protocols in commercial spinach production systems in saline soils, contributing to both food security and agricultural sustainability goals.

## Supplementary Information

Below is the link to the electronic supplementary material.

Supplementary Material 1

## Data Availability

The data of current study are available from the corresponding author on reasonable request.
