# Supplementary material for: Selenium biofortification in conditions of soil salinity improve some nutritional value parameters of spinach for the fresh market
Source: Sci Rep. 2026 Jun 12;16:26901. doi: 10.1038/s41598-026-55268-4 (PMC13518849; doi:10.1038/s41598-026-55268-4)
Supplement: Supplementary file 1 [file 41598_2026_55268_MOESM1_ESM.docx]

**Selenium biofortification in conditions of soil salinity improve some nutritional value parameters of spinach for the fresh market**

**Ewelina Grochowska^1^*, Marcin Niemiec^1^, Monika Komorowska^1^, Monika Kula-Maximenko^2^,** **Suat Hian Tan^3^, Abzal Abdramanov^4^, Anna Gorczyca^5^***

^1^ Department of Agricultural and Environmental Chemistry, ^5^ Department of Microbiology and Biomonitoring, Faculty of Agriculture and Economics, University of Agriculture in Krakow, Mickiewicza 21, 31-120 Krakow, Poland, e-mail: ewelina.grochowska@urk.student.edu.pl, https://orcid.org/0009-0004-6754-0244; marcin.niemiec@urk.edu.pl, https://orcid.org/0000-0001-9144-3728; monika.komorowska@urk.edu.pl; anna.gorczyca@urk.edu.pl, https://orcid.org/0000-0002-9431-3870

^2^ Franciszek Górski Institute of Plant Physiology Polish Academy of Sciences, Niezapominajek 21, 30-239 Krakow, Poland, e-mail: m.kula@ifr-pan.edu.pl, https://orcid.org/0000-0001-7100-877X

^3^ Faculty of Industrial Sciences & Technology, Universiti Malaysia Pahang, Gambang, 26300, Pahang, Malaysia, e-mail: tshian@ump.edu.my, https://orcid.org/0000-0001-8035-9109

^4^ Kazakh National Agrarian Research University, 8, Abay av. 050010 Almaty, Kazakhstan, e-mail: abzal.abdramanov@kaznaru.edu.kz, https://orcid.org/0000-0002-6268-3340

* Corresponding author: Ewelina Grochowska; e-mail: ewelina.grochowska@urk.student.edu.pl;

Anna Gorczyca; anna.gorczyca@urk.edu.pl

**Meteorological conditions during the spinach growing season**

Average daily air and soil temperature, deltaT (wet and dry bulb temperature difference)
and dew point

Relative humidity and sum of daily precipitation

,

Wind flow speed, solar radiation and ET0 (sum of water evaporation from the soil and water transpiration through plants)

**Verification of the assumptions of normality of the residual distribution (Shapiro-Wilk) and homogeneity of variance (Levene)**

| **Parametr** | **Shapiro-Wilka test (p)** | **Levene'a test (p)** |
| --- | --- | --- |
| Yield | 0.187 | 0.342 |
| Dry mass | 0.234 | 0.451 |
| Chl *a* | 0.412 | 0.298 |
| Chl *b* | 0.156 | 0.387 |
| Car | 0.089 | 0.123 |
| Phenolic | 0.203 | 0.415 |
| Ascorbic acid | 0.178 | 0.298 |
| Antioxidant activity | 0.112 | 0.187 |
| Cl | 0.065 | 0.234 |
| K | 0.231 | 0.356 |
| S | 0.098 | 0.421 |
| Na | 0.145 | 0.298 |
| Se | 0.067 | 0.156 |

*For all tested parameters, the p values for both tests exceed 0.05, which means that the assumptions of the linear model are met.*

**ANOVA Two-Way**

**fresh leaf biomass yield**

**dry matter bioaccumulation**

**chlorophyll *a***

**chlorophyll *b***

**carotenoids**

**phenols**

**L-ascorbic acid**

**Antioxidant activity**

**chlorine content**

**potassium content**

**sulfur content**

**sodium content**

**selenium content**

**Se biofortification efficiency coefficient as a percentage of Se taken by the crop to the total amount of selenium used in foliar application**

| **Object designation** | **abbreviation** | **Se recovery efficiency [%]** |
| --- | --- | --- |
| selenium low dose | SeL | 34.30 |
| selenium high dose | SeH | 22.49 |
| low salt stress plus selenium low dose | SLSeL | 35.18 |
| low salt stress plus selenium high dose | SLSeH | 24.24 |
| high salt stress plus selenium low dose | SHSeL | 35.71 |
| high salt stress plus selenium high dose | SHSeH | 20.93 |
